# Dosimetric Predictors of Acute and Chronic Alopecia in Primary Brain Cancer Patients Treated With Volumetric Modulated Arc Therapy

**DOI:** 10.3389/fonc.2020.00467

**Published:** 2020-04-08

**Authors:** Silvia Scoccianti, Gabriele Simontacchi, Daniela Greto, Marco Perna, Francesca Terziani, Cinzia Talamonti, Maria Ausilia Teriaca, Giorgio Caramia, Monica Lo Russo, Emanuela Olmetto, Camilla Delli Paoli, Roberta Grassi, Vincenzo Carfora, Calogero Saieva, Pierluigi Bonomo, Beatrice Detti, Monica Mangoni, Isacco Desideri, Giulio Francolini, Vanessa Di Cataldo, Livia Marrazzo, Stefania Pallotta, Lorenzo Livi

**Affiliations:** ^1^Radiation Oncology Unit, Azienda Ospedaliera Universitaria Careggi, University of Florence, Florence, Italy; ^2^Medical Physics Unit, Department of Experimental and Clinical Biomedical Sciences “Mario Serio,” Azienda Ospedaliera Universitaria Careggi, University of Florence, Florence, Italy; ^3^SC Epidemiology of Risk Factors and Lifestyles, Institute for Study, Prevention, and Oncology Network (ISPRO), Florence, Italy

**Keywords:** alopecia, radiation-induced hair loss, scalp, constraints, predictors, VMAT, brain tumors, radiotherapy

## Abstract

**Purpose:** To determine dose constraints that correlate with alopecia in patients treated with photon-based Volumetric Modulated Arc Therapy (VMAT) for primary brain tumors.

**Methods:** During the treatment planning process, the scalp was drawn as a region of interest. Dose received by 0.1 cc (D_0.1cc_), mean dose (D_mean_), absolute volumes receiving different doses (V_16Gy_, V_20Gy_, V_25Gy_, V_30Gy_, V_35Gy_, V_40Gy_, and V_43Gy_) were registered for the scalp. Alopecia was assessed according to Common Terminology Criteria for Adverse Events (CTCAE) v4.0. Receiver operating characteristics (ROC) curve analysis was used to identify parameters associated with hair-loss.

**Results:** One-hundred and one patients were included in this observational study. At the end of radiotherapy (RT), 5 patients did not develop alopecia (D_mean_ scalp 3.1 Gy). The scalp of the patients with G1 (*n* = 11) and G2 (*n* = 85) alopecia received D_mean_ of 10.6 Gy and 11.8 Gy, respectively. At ROC analysis, V_16Gy20Gy_ ≥ 5.2 cc were the strongest predictors of acute alopecia risk. Chronic hair-loss assessment was available for 74 patients: median time to recovery from G2 alopecia was 5, 9 months. The actuarial rate of hair regrowth was 98.1% at 18 months after the end of RT. At ROC analysis, V_40Gy43Gy_ ≥2.2 cc were the strongest predictors of chronic G2-alopecia risk. V_20Gy_, V_40Gy_, and D_0,1cc_ were shown to be independent variables according to correlation coefficient r.

**Conclusions:** V_20Gy_ and V_40Gy_ were the strongest predictors for acute and chronic G2 hair-loss, respectively. The low-dose bath typical of VMAT corresponds to large areas of acute but transient alopecia. However, the steep dose gradient of VMAT allows to reduce the areas of the scalp that receive higher doses, minimizing the risk of permanent alopecia.

The application of our dosimetric findings for the scalp may help in reducing the alopecia risk and also in estimating the probability of hair-loss during patient counseling before starting radiotherapy.

## Introduction

Due to the high radiosensitivity of hair follicles, radiotherapy (RT) may induce hair-loss with a huge psychological impact and, thus, negative effects on patient's quality of life, also in case of limited life expectancy (1–5).

In the treatment of brain tumors, the technology of IMRT and, most recently, rotating gantry IMRT techniques such as VMAT, can produce dose distributions that conform to the target volume and deliver a reduced dose to the critical organs (6).

Recently, due to the increased conformality of IMRT techniques, there has been considerable interest in sparing critical structures not classically included into the list of intracranial organs at risk, such as hippocampus (7) or dorsal vagal complex (8). Likewise, the inclusion of the scalp among the organs at risk may potentially reduce the incidence or the severity of hair loss.

In the present study we included a total of 101 patients whose scalp was drawn as a region of interest to spare during the treatment planning process. The present work reports a dosimetric analysis of the scalp describing the risk of acute and permanent hair-loss following cranial irradiation on limited volume, performed with a VMAT approach.

The primary objective is to define dosimetric predictors for hair-loss with the aim of using them as dose constraints during the inverse planning process. Secondary aims were to analyze the recovery time and to evaluate clinical factors possibly associated with permanent alopecia.

## Methods and Materials

Consecutive patients treated for a primary brain tumor in our Institute with a conventionally fractionated VMAT were included in this observational study. Eligibility criteria included the use of partial brain radiotherapy, conventional fractionation, total dose >50 Gy, life expectancy > 4 months. Exclusion criteria included previous radiation treatment on the brain; previous chemotherapy; the need for whole brain radiotherapy; any previously existing alopecia according to Basic and specific (BASP) classification (9). All patients signed a consent form before enrollment in this institutional review board-approved study. Factors that may have an impact on alopecia such as age, smoking history, use of antiepileptic drugs (AEDs) and chemotherapy were registered.

### Scalp as a Region of Interest During the Treatment Planning Process

CT (Computed Tomography) image sets for radiation treatment planning were acquired using a Brilliance Big Bore CT (Philips Medical Systems). The slice thickness was 2 mm.

During the contouring process, a region of interest (ROI) was defined for the scalp.

**Figure 1:**
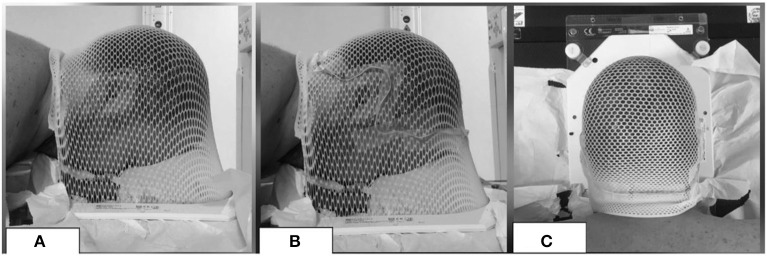
Example of a mask in prone position **(A)** with a wire **(B,C)** to exclude the hairless skin from the ROI of the scalp.

At the moment of simulation CT, beyond the custom thermoplastic mask with the patient in the supine position (used as immobilization device during the treatment, as usual), for each patient a mask in prone position was molded (Figure 1A). With the aim of tracing the extension of the follicle-bearing scalp, the line between the hairy scalp and the hairless skin of the face and of the neck was defined with a wire (Figures 1B,C). CT scan of the mask in prone position without the patient was acquired for each case. These images were co-registered to the simulation CT of the corresponding patient, in order to avoid the hairless skin beyond the wire.

**Figure 2:**
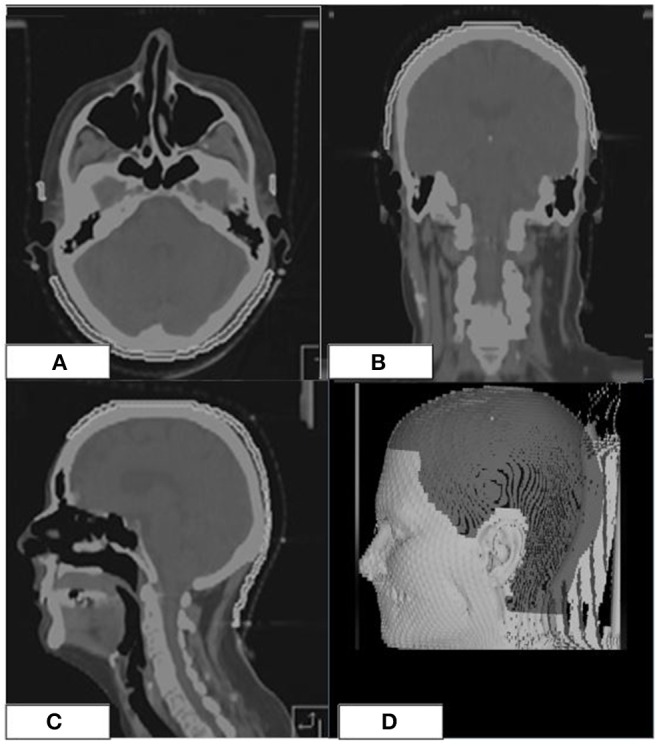
Scalp ROI (white line) on the simulation CT of a patient **(A–C)**. 3D-view showing the scalp ROI **(D)**.

The scalp volume was defined as a ROI including the hair-bearing tissue between the skin and the outer table of the skull, up to a maximum thickness of 5 mm (4, 10–12) (Figure 2).

The definition of the clinical target volume (CTV) varied according to the primary tumor. The planning target volume (PTV) was generated by adding a 3 mm isotropic margin to CTV.

VMAT plans were generated with Monaco (CMS-Elekta Ltd, UK) using a Monte-Carlo algorithm. Most of the cases were treated with a coplanar-partial arc technique. During the treatment planning, the scalp dose was kept as minimal as possible. Constraints to the other intracranial organs at risk [brainstem, optic chiasm and nerves, cochleas, pituitary (13)] had a higher priority than those of the scalp. The irradiation was delivered, using 6-MV photons with an Elekta Synergy machine equipped with a Beam Modulator multi-leaf collimator.

### Hair Loss Assessment

Alopecia was assessed according to CTCAE version 4.0: G1 alopecia was defined as hair-loss of <50% of normal for that individual that is not obvious from a distance but only on close inspection; a different hairstyle may be required to cover the hair loss but it does not require a wig or hairpiece to camouflage; G2 alopecia was defined as hair-loss of ≥50% normal for that individual that is readily apparent to others; a wig or hairpiece was necessary if the patient desires to completely camouflage the hair loss; associated with psychosocial impact.

**Figure 3:**
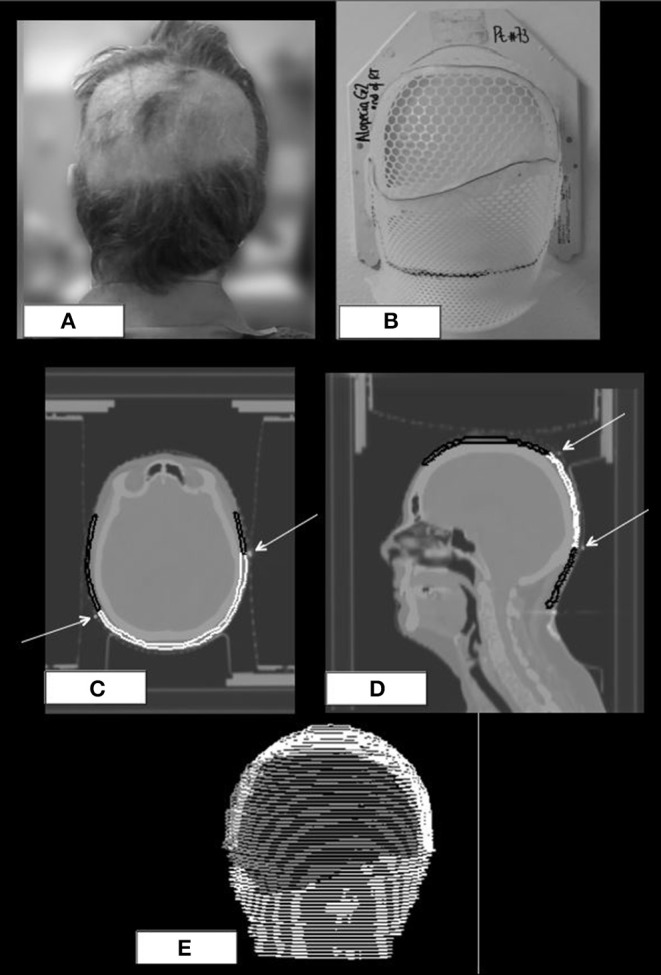
Posterior view of a patient with a wide area of G2 alopecia **(A)** at the end of radiotherapy; prone mask with the wire defining the area of alopecia **(B)**; original CT simulation of the patient with coregistration of the wired prone mask **(C,D)**; scalp ROI is black colored, area of alopecia G2 is white colored, white arrows indicate the wire on the mask. 3D-view of the same patient at the treatment planning **(E)**.

At the end of radiotherapy, in order to define the exact extension of the areas of acute alopecia, patients were required to wear the prone mask that had been molded during the simulation CT. Areas of alopecia were defined on the mask with a wire (Figures 3A,B); a new CT of the mask without the patient was acquired and, then, co-registered with the original simulation CT of the corresponding patient. Afterwards, areas of alopecia were contoured in order to obtain a treatment planning system-based dosimetric evaluation of the acute hair loss areas (Figures 3C,E).

For all the patients dose-volume histograms of the following ROIs were created: whole scalp, areas where G1 alopecia had developed during the treatment (G1-alopecia_endof RT_), areas where G2 alopecia had developed during the treatment (G2- alopecia_endofRT_).

Data regarding volumes in cc were collected both for the whole scalp and for the areas of acute alopecia. The following dosimetric parameters were collected: dose received by 0.1 cc of the ROI (D_0.1cc_), mean dose (D_mean_), absolute volumes that received 16, 20, 25, 30, 35, 40, and 43 Gy (V_16Gy_, V_20Gy_, V_25Gy_, V_30Gy_, V_35Gy_, V_40Gy_, and V_43Gy_).

Patients were evaluated for hair loss at the end of radiotherapy and, then, every 3 months for the first 3 years of follow up.

G2 hair loss persisting for >9 months after the end of RT was defined as chronic alopecia.

### Statistical Methods

A comparison between the dosimetric data was performed with the Mann–Whitney test.

The probability of developing acute G2 alopecia as a function of the D_0,1cc_ was calculated using the maximal likelihood method according to the formula *P*(D) = [1 + (D_50_/D)^4γ50^]^−1^, where the D_50_ was D_0,1cc_ at which 50% of the patients developed acute alopecia and γ50 was the slope of the curve.

Receiver operating characteristics ROC analysis (14, 15), already adopted by other authors to identify dosimetric parameters associated with RT damage (16, 17), was used to identify the dosimetric parameters related to the risk of G2 alopecia. The maximum value of the Youden index (J) (18) was used for selecting the optimal cut-off point for each dosimetric variable.

Intercorrelation between dosimetric factors was analyzed: dosimetric variables with coefficient *r* < 0.75 were considered independent predictors.

Impact of clinical factors on incidence of acute alopecia was analyzed with chi-squared (χ2) test.

Kaplan–Meier survival analysis was carried out concerning alopecia recovery. The observation time was measured from the end of radiotherapy to complete recovery from alopecia or to the last follow-up for cases with the persistence of hair-loss. Differences between groups were evaluated by the log-rank test. Cox proportional regression analysis was used to determine the role of selected parameters on the risk of event occurrence by univariate models. Multivariate Cox proportional- hazards regression analysis was performed including only the variables that were shown to be not intercorrelated (coefficient *r* < 0.75).

All the statistical tests were performed using the IBM-SPSS Statistics software (Statistical Package for Social Science, version 22).

## Results

A total of 101 patients were included in the study. Characteristics of the patients are in Table 1.

**Table 1:** Patients characteristics.

		***n***	**Proportion (%)**
Patients	All	101	100
Gender	Female	48	47.5
	Male	53	52.5
Age	Mean 51.7		
	Age <14	4	4.0
	Age ≥ 14	97	96.0
	Age <50	38	37.6
	Age ≥ 50	63	62.4
Smoking history	no	68	67.3
	yes	33	32.7
Histology	High grade gliomas	68	67.3
	Low grade gliomas	12	11.9
	Meningioma	10	9.9
	Others	11	10.9
Antiepilectic drugs during radiotherapy	no	24	23.8
	yes	77	76.2
Concomitant chemotherapy	no	41	40.6
	Temozolomide	60	59.4
Chemotherapy after radiotherapy	no	34	33.7
	Temozolomide	59	58.4
	Procarbazine, vincristine, lomustine	8	7.9

Prescription doses ranged between 50.4 and 60 Gy in conventional fractionation. Mean scalp volume was 234.8 cc (SD 46.9).

Dosimetry of the whole scalp was available for all the patients. Of note, among the dosimetric parameters whose values were collected, V_20Gy_, V_40Gy_, and D_0,1cc_ were shown to be independent variables according to correlation coefficient r.

### Acute Alopecia

Clinical and dosimetric evaluation at the end of RT was available for all the patients.

### Acute Alopecia: Dosimetry of the Whole Scalp

Five patients who were treated for deep tumors (pituitary adenomas *n* = 4; parasellar meningioma *n* = 1) did not develop any area of alopecia. The remaining 96 patients developed acute alopecia: 11 developed G1 alopecia only whereas 85 patients developed G2 alopecia (G2 only *n* = 52; G1+G2 *n* = 33). Significant differences in the dosimetric parameters were found between the scalp of the patients who did not develop alopecia and the scalp of patients who developed acute G1 alopecia and G2 alopecia (Table 2).

**Table 2:** Dosimetric comparison between the scalp of the patients who did not develop alopecia and the scalp of the patients who developed G1 or G2 alopecia at the end of radiotherapy: mean values and standard deviations (in brackets) of dosimetric variables.

	***n***	**Mean D_**0.1cc**_ (Gy)**	**Mean D_**mean**_ (Gy)**	**Mean V_**16Gy**_ (cc)**	**Mean V_**20Gy**_ (cc)**	**Mean V_**25Gy**_ (cc)**	**Mean V_**30Gy**_ (cc)**	**Mean V_**35Gy**_ (cc)**	**Mean V_**40Gy**_ (cc)**	**Mean V_**43Gy**_ (cc)**
Scalp_patients no alopecia_	5	19.7 (± 12.6)	3.1 (± 1.5)	2.7 (± 3.7)	1.4 (± 1.9)	0.6 (± 1.1)	0.2 (± 0.5)	0.04 (± 0.09)	0	0
Scalp_patients with G1 alopecia_ _at the end of RT_	11	40.2 (± 15.2)	10.6 (± 5.0)	45.2 (± 40.4)	31.0 (± 33.9)	22.2 (± 26.9)	15.6 (± 20.5)	10.8 (± 15.6)	7.4 (± 12.1)	5.8 (± 10.0)
*p-value from Mann–Whitney Test*		**0.02**	**0.001**	**0.002**	**0.02**	**0.03**	**0.04**	0.90	0.90	0.09
Scalp_patients with G2 alopecia_ _*at the end of RT*_	85	47.3 (± 9.2)	11.8 (± 4.4)	68.8 (± 37.7)	50.6 (± 33.4)	34.8 (± 27.5)	23.1 (± 22.0)	14.3 (± 16.9)	8.4 (± 12.0)	5.9 (± 9.3)
*p-value from Mann-Whitney Test*		**0.0001**	**0.0001**	**0.0001**	**0.0001**	**0.0001**	**0.0001**	**0.001**	**0.004**	**0.005**

*D_0.1cc_, dose received by 0.1 cc; D_mean_, mean dose; V_xGy_, percentage of the scalp volume receiving ≥ x Gy*.

*Bold text indicates statistical significance*.

**Figure 4:**
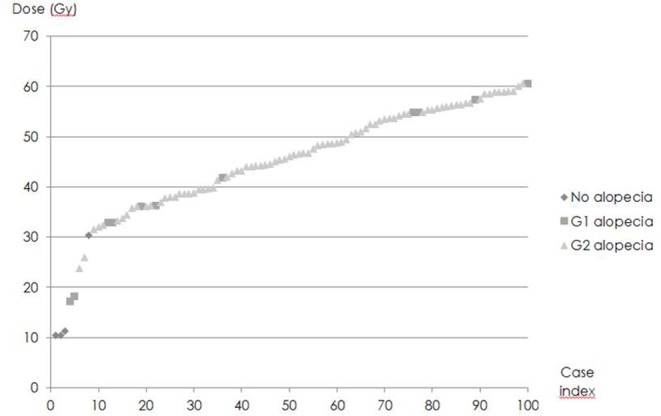
Distribution of Maximum dose (D_0,1cc_) in the series and grade of acute alopecia.

**Figure 5:**
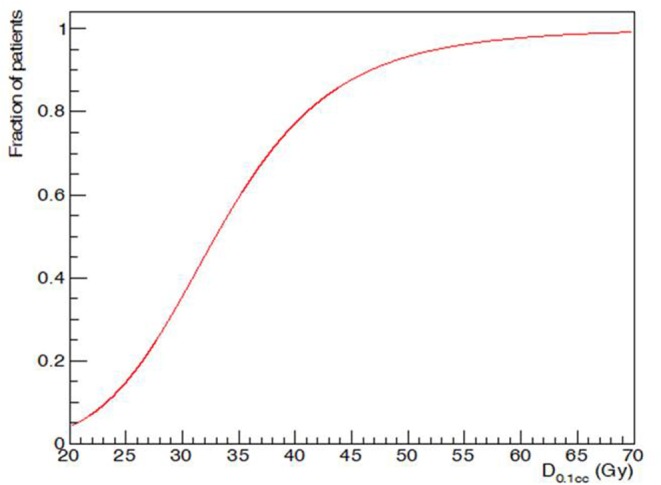
Maximum dose (D_0,1cc_) and acute G2 alopecia probability at the end of radiotherapy: dose-response relationship.

D_0,1cc_ varied widely (Figure 4). D_50_, i.e., D_0,1cc_ at which 50% of the patients developed acute alopecia was found to be 33,0 ± 0,2 Gy. The slope of the curve (γ50) was 1,58 ± 0,05 (Figure 5).

### Acute Alopecia: Dosimetry of the Areas of Alopecia

**Figure 6:**
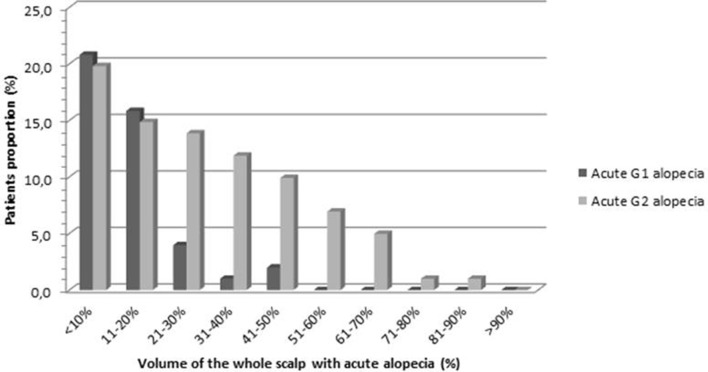
Percentage of the volume of the scalp with G1 and G2 alopecia at the end of radiotherapy.

Volumetric data regarding the areas of alopecia were collected in order to define the amount of hair loss in terms of percentage of the scalp volume at the end of radiotherapy (Figure 6). The mean volume of G1-alopecia_end−of−RT_ and G2-alopecia_end−of−RT_ was 26.6 and 66.1 cc, respectively. On average, G1-alopecia_end−of−RT_ and G2-alopecia_end−of−RT_ corresponded to 11.9% (SD 10.4) and 41.7% (SD 20.0) of the whole scalp volume, respectively. The mean volume of alopecia of any grade was 70.7 cc (corresponding to 30.2% of the scalp, SD 20.7)

Significant differences in the dosimetric parameters were found when G1-alopecia_end−of−RT_ were compared with alopecia G2_end−of−RT_ (Table 3).

**Table 3:** Dosimetric comparison between the areas of G1 alopecia and the areas of G2 alopecia at the end of radiotherapy: mean values and standard deviations (in brackets) of dosimetric variables.

	***n***	**Mean D_**0.1cc**_ (Gy)**	**Mean D_**mean**_ (Gy)**	**Mean V_**16Gy**_ (cc)**	**Mean V_**20Gy**_ (cc)**	**Mean V_**25Gy**_ (cc)**	**Mean V_**30Gy**_ (cc)**	**Mean V_**35Gy**_ (cc)**	**Mean V_**40Gy**_ (cc)**	**Mean V_**43Gy**_ (cc)**
G1-Alopecia_end of RT_	44	33.4 (± 14.3)	16.5 (± 8.3)	11.9 (± 14.6)	7.6 (± 9.6)	6.5 (± 11.8)	3.8 (± 7.9)	2.4 (± 6.4)	1.6 (± 5.4)	1.3 (± 4.8)
G2-Alopecia_end of RT_	85	44.6 (± 11.2)	20.3 (± 6.4)	40.2 (± 35.4)	31.5 (± 30.5)	21.8 (± 24.3)	14.8 (± 18.9)	9.2 (± 14.3)	5.4 (± 10.2)	3.6 (± 7.7)
*p-value from Mann-Whitney Test*		**0.0001**	**0.002**	**0.0001**	**0.0001**	**0.0001**	**0.0001**	**0.001**	**0.0001**	**0.001**

*D_0.1cc_, dose received by 0.1 cc; D_mean_, mean dose; V_xGy_, percentage of the scalp volume receiving ≥ x Gy*.

*Bold text indicates statistical significance*.

### Acute Alopecia: ROC Analysis

At ROC analysis, all the dosimetric variables were found to be reliable parameters to distinguish patients at low-risk from those at high-risk of acute G2 alopecia (Table 4).

**Table 4:** Receiver operating characteristics (ROC) analysis for G2 alopecia at the end of radiotherapy.

**Dosimetric variable**	**AUC**	***p*-value for AUC**	**Cut-off value**	**Sensitivity, %**	**Specificity, %**	**Incidence of acute G2 alopecia**		**Fisher exact test *p*-value**
						**Low risk, %**	**High risk, %**	
D_0.1cc_	0.740	**0.008**	*36.2 Gy*	87.1	68.7	50.0%	93.7%	**0.0000**
Mean dose	0.714	**0.02**	*6.9 Gy*	87.1	56.2	55.0%	91.3%	**0.0006**
V_16Gy_	0.776	**0.001**	*16.7 cc*	92.9	62.5	37.5%	92.9%	**0.0000**
V_20Gy_	0.792	**0.0003**	*5.2 cc*	100.0	56.2	0%	91.4%	**0.0000**
V_25Gy_	0.768	**0.002**	*5.5 cc*	85.9	68.7	52.2%	93.5%	**0.0000**
V_30Gy_	0.756	**0.003**	*2.3 cc*	85.9	68.7	52.2%	93.6%	**0.0000**
V_35Gy_	0.736	**0.005**	*0.7 cc*	82.4	68.7	57.7%	93.3%	**0.0005**
V_40Gy_	0.685	**0.02**	*0.6 cc*	72.9	75.0	65.7%	93.9%	**0.0004**
V_43Gy_	0.670	**0.04**	*0.1 cc*	70.6	68.7	69.4%	92.3%	**0.004**

*D_0.1 cc_, Dose to 0.1 cc of the scalp volume; V_xGy_, percentage of the scalp volume receiving ≥ x Gy; AUC, area under curve at ROC analysis*.

*Bold text indicates statistical significance*.

V_16Gy_ and V_20Gy_ were found to be the strongest predictors for acute alopecia (AUC 0.776 and 0.792, respectively). Cut-off values for high risk of development of alopecia at the end of radiation treatment were 16.7 cc and 5.2 cc for V_16Gy_ and V_20Gy_, respectively.

### Factors Impacting on Acute Alopecia

Gender (χ2 test: *p* = 0.19), age (χ2 test: *p* = 0.37), smoking history (χ2 test: *p* = 0.65), use of AEDs (χ2 test: *p* = 0.09), concomitant chemotherapy (χ2 test: *p* = 0.17) did not have any significant impact on acute hairloss incidence.

### Chronic Alopecia

All the cases of persistent alopecia were an evolution of acute alopecia (i.e., all the patients who had chronic alopecia, had had previous acute alopecia in the same areas that did not recover; on the contrary, all the patients who had had no acute alopecia (n = 5) did not develop chronic alopecia).

Hair-loss assessment for G2-alopecia was available for 74 patients. The mean follow-up was 9.7 months. At the moment of analysis, 65/74 (87.8%) patients had a complete G2 recovery.

**Figure 7:**
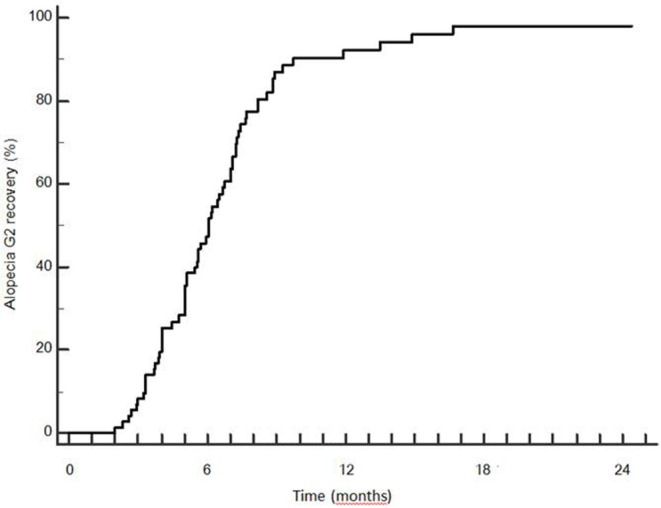
Time to recovery from G2 alopecia.

Late recovery from G2 hairloss was possible: 3 patients recovered between 12 and 18 months. Median time to recovery was 5.9 months (SD 2.8 months). Actuarial rate of G2 recovery was 49.2, 87.0, 92.2, and 98.1% at 6, 9, 12, and 18 months after the end of RT (Figure 7).

### Chronic Alopecia: Dosimetry of the Whole Scalp

Dosimetric analysis of the whole scalp excluded patients with a follow-up shorter than 3 months (*n* = 5). Dosimetric parameters of the whole scalp of 8 patients who had a persistent alopecia (>9 months) were compared with the dosimetric data of 66 patients who had an intact scalp within 9 months after the end of RT (Table 5). Of note, patients who had not developed alopecia at the end of radiotherapy (*n* = 5) were included among these 66 patients. V_40Gy_ and V_43Gy_ were statistically different between these two groups (Mann–Whitney test *p* = 0.028 and *p* = 0.036).

**Table 5:** Dosimetric comparison of the scalp of the patients who had persistent alopecia at 9 months compared with the scalp of the patients who had complete recover within 9 months: mean values and standard deviations (in brackets) of dosimetric variables.

**ROI**	***n***	**Mean D_**0.1cc**_ (Gy)**	**Mean D_**mean**_ (Gy)**	**Mean V_**16 Gy**_ (cc)**	**Mean V_**20 Gy**_ (cc)**	**Mean V_**25 Gy**_ (cc)**	**Mean V_**30 Gy**_ (cc)**	**Mean V_**35 Gy**_ (cc)**	**Mean V_**40 Gy**_ (cc)**	**Mean V_**43 Gy**_ (cc)**
Scalp _patients with alopecia at 9 month−follow up_	8	53.2 (± 4.1)	14.1 (± 4.4)	78.7 (± 31.3)	57.8 (± 25.0)	40.1 (± 17.4)	28.4 (± 12.7)	19.6 (± 9.7)	12.6 (± 8.4)	8.7 (± 7.6)
Scalp _patients with complete recovery from_ _alopecia within 9 months after RT_	66	46.7 (± 10.6)	11.6 (± 4.7)	66.8 (± 39.3)	49.5 (± 35.4)	34.7 (± 29.9)	23.4 (± 24.3)	14.8 (± 18.7)	8.9 (± 13.3)	6.4 (± 10.4)
*p-value from Mann-Whitney Test*		0.12	0.09	0.29	0.34	0.24	0.14	0.053	**0.028**	**0.036**

*D_0.1cc_, dose received by 0.1 cc; D_mean_, mean dose; V_xGy_, percentage of the scalp volume receiving ≥ x Gy*.

*Bold text indicates statistical significance*.

### Chronic Alopecia: ROC Analysis

At ROC analysis, several dosimetric variables were significantly related to the risk of permanent alopecia (Table 6). Among these, V_40Gy_ and V_43Gy_ were the strongest predictors for chronic G2-alopecia (AUC = 0.738 and 0.725, respectively): patients whose scalp ROI had V_40Gy43Gy_.

**Table 6:** Receiver operating characteristics (ROC) analysis for G2 alopecia at 9 months after the end of radiotherapy.

**Dosimetric variable**	**AUC**	***p*-value for AUC**	**Cut-off**	**Sensitivity, %**	**Specificity, %**	**Incidence of G2 alopecia at 9 months**		**Fisher exact test *p*-value**
						**Low risk, %**	**High risk, %**	
D_0.1cc_	0.684	**0.008**	*47.6 Gy*	100.00	51.5	0.0%	17.4%	**0.007**
Mean dose	0.669	**0.04**	*10.1 Gy*	100.00	43.9	0.0%	14.0%	**0.02**
V_30Gy_	0.662	**0.04**	*11.4 cc*	100.00	39.4	0.0%	13.1%	**0.04**
V_35Gy_	0.710	**0.001**	*9.3 cc*	100.00	54.5	0.0%	17.4%	**0.005**
V_40Gy_	0.738	**<0.0001**	*5.4 cc*	100.00	63.64	0.0%	21.0%	**0.0000**
V_43Gy_	0.725	**0.0002**	*2.2 cc*	100.00	59.1	0.0%	19.5%	**0.001**

*D_0.1cc_, Dose to 0.1 cc of the scalp volume;V_xGy_, percentage of the scalp volume receiving ≥ x Gy; AUC, area under curve at ROC analysis*.

*Bold text indicates statistical significance*.

### Kaplan–Meier Analysis and Cox Regression for Recovery From Alopecia

All the dosimetric parameters that were found to be significant predictors of chronic G2-alopecia at the ROC analysis (D_0.1cc_, D_mean_, V_30Gy_, V_35Gy_, V_40Gy_, and V_43Gy_) and all the clinical variables were included in the Kaplan–Meier analysis (Table 7).

**Table 7:** Kaplan Meier analysis for factors impacting on the recovery probability from G2 alopecia.

**Variable**	**pts**	**Events**	**%recovery**	***p*-value log rank test**
**Sex**				
F	33	28	–	0.99
M	41	37		
**Age**				
≤ 14	4	4	100.0	**0.01**
>14	70	61	97.9	0.70
≤ 50	30	28	–	
>50	44	37		
**Smoking history**				
No	51	46	–	0.54
Yes	23	19		
**Antiepilectic drugs**				
No	17	16	–	0.14
Yes	57	49		
**Concomitant chemotherapy**				
No	25	23	–	0.14
Yes	49	42		
**Sequential chemotherapy**				
No	21	19	–	0.17
Yes	53	46		
**D**_**0.1 cc**_				
<47.6 Gy	34	30	99.9	**0.001**
≥47.6 Gy	40	35	96.6	
**D**_**mean**_				
<10.1 Gy	29	27	99.9	**0.002**
≥10.1 Gy	45	38	96.7	
**V**_**30 Gy**_				
<11.4 cc	26	24	99.9	**0.0001**
≥11.4 cc	48	41	97.0	
**V**_**35 Gy**_				
<9.3 cc	36	32	99.9	**0.0001**
≥9.3 cc	38	33	96.4	
**V**_**40 Gy**_				
<5.4 cc	42	38	99.9	**0.0001**
≥5.4 cc	32	27	95.6	
**V**_**43 Gy**_				
<2.2 cc	34	34	99.9	**0.0001**
≥2.2 cc	40	31	96.9	
Total	*74*	*65*		

*Bold text indicates statistical significance*.

Patients were stratified according to the cut-off values defined at the ROC analysis for each dosimetric variable with the aim to have dichotomous variables. All the tested dosimetric parameters significantly impacted on recover probability.

Age had a significant impact on recover probability (age > 14 = 97.9 vs. age ≤ 14 = 100%; log-rank test *p* = 0,01). No other clinical factors (gender, smoking history, use of AEDs, chemotherapy) significantly influenced the recover probability. Impact on recover probability due to different chemotherapy schedules was not tested because, among the cases with trichological follow-up, nearly all patients who had sequential chemotherapy received temozolomide (52 out of the 53).

Age and all the above mentioned dichotomous dosimetric variables were found to be significant at univariate Cox regression (Table 8). D_0,1cc_ maintained significance also when tested as a continuous variable (*p* = 0,001) at the univariate analysis.

**Table 8:** Univariate Cox regression for variables impacting the recovery probability from G2 alopecia.

**Variable**	***p*-value**	**HR**	**95% CI**
Age > 14 y	**0.017**	0.27	0.09–0.80
D_**0.1 cc**_ > 47.6 Gy	**0.001**	0.40	0.23–0.69
D_**mean**_ > 10.1 Gy	**0.003**	0.43	0.24–0.75
V_**30Gy**_ > 11.4 cc	**0.0001**	0.39	0.22–0.67
V_**35Gy**_ > 9.3 cc	**0.0001**	0.33	0.19–0.57
V_**40Gy**_ > 5.4 cc	**0.0001**	0.35	0.20–0.63
V_**43Gy**_ > 2.2 cc	**0.0001**	0.36	0.21–0.64

*D_0.1 cc_, Dose to 0.1 cc of the scalp volume;V_xGy_, percentage of the scalp volume receiving ≥ x Gy; HR, Hazard Ratio; CI, Confidence interval. Bold text indicates statistical significance*.

Among the dosimetric factors, only V_40Gy_ and D_0,1cc_ were included in the multivariate Cox regression, because they were shown to be independent predictors of chronic G2-alopecia according to correlation coefficient r. Multivariate analysis confirmed the predictive value of age (*p* = 0.0002) and V_40Gy_ (*p* = 0.02).

## Discussion

Hair loss, either temporary or permanent, is one of the most stressful side effects for patients undergoing oncologic treatment (1–5). Radiation-induced alopecia may permanently alter the self-perception of the neurooncological patients and have a significant impact on their quality of life (2, 5).

To our knowledge, this is the first study reporting a dose-volume analysis of the scalp describing the risk of hair-loss following a photon-based, conventionally fractionated VMAT treatment on a limited brain volume. Herein we reported a dosimetric analysis based on a TPS-based calculation to find a dose-response relationship for acute and chronic alopecia. Besides, although some authors reported about the possibility of hair regrowth within some months after irradiation (19, 20), to our knowledge, this is the first observational study focusing on the analysis of recovery time of the scalp damage.

### Dose-Response Relationship for Acute Alopecia

On review of the available literature regarding photon-based radiotherapy, the doses that have been reported to cause hair-loss varied widely. Doses as low as 2 and 3 Gy in a single fraction might cause temporary alopecia according to some authors (4, 10, 21, 22). In a study regarding the use of VMAT for whole-brain irradiation (WBRT) in patients with multiple brain metastases (19), the authors hypothesized that the threshold dose for temporary alopecia is around 10 Gy in 5 fractions. By contrast, Archambeau et al. (23) described that acute epilation may be produced by a total dose of 20 Gy in conventional fractionation.

The risk of acute alopecia during IMRT has been explored also for patients with head and ncek cancer: Rosenthal et al. (24) provided recursive partitioning analysis in order to estimate dose thresholds associated with observed toxicities in a series of patients with oropharyngeal cancer treated with IMRT: they found that alopecia in the occipital region occurred more frequently when scalp maximum dose was >30 Gy (48% of cases) vs. <30 Gy (19% of cases).

Our experience confirmed that acute alopecia may be caused by very low doses: acute G2 alopecia developed also in areas where D_mean_ may be as low as 1.9 Gy.

However, the fact that we found significant differences regarding the dose received by the whole scalp between patients that did not develop alopecia and patients who presented acute hair-loss, demonstrated that a dose relationship with acute alopecia exists. That was also confirmed by the dosimetric analysis regarding the areas of alopecia: G2-alopecia_end−of−RT_ received significantly higher doses than G1-alopecia_end−of−RT_ (Table 3). Lastly, the relationship between dose and acute alopecia was also evidenced by the ROC analysis that showed that the most important predictors of acute alopecia were V_16Gy_ and V_20Gy_ (Table 4).

Consequently, during the treatment planning process, the doses to the scalp should be kept as low as possible. However, by maintaining V_16Gy_ < 16.7 cc and V_20Gy_ < 5.2 cc, the risk of acute alopecia may be limited. Since these two variables were found to be interdependent, considering the better AUC and statistical significance at the ROC analysis, we would suggest to try to meet preferably the specified constraints for V20, with the aim of reducing the risk of acute alopecia.

Moreover, we also found that 50% of the patients who received D_0.1cc_ of 33 Gy developed acute alopecia at the end of radiotherapy. All these data may be precious to predict the risk of acute hairloss when we talk with the patients about the toxicity of the radiation treatment.

### Dose-Response Relationship for Chronic Alopecia

Our data showed that a dose-effect relationship exists for chronic alopecia as well: the scalp of patients who completely recovered from G2 alopecia received lower doses than the scalp of patients who had persistent alopecia at 9 months. Of note, the difference between these two groups of patients was significant only in terms of high doses (V_40Gy_, V_43Gy_) (Table 6). Noteworthy, at ROC analysis lower doses (<30 Gy) were not associated with chronic G2-alopecia, while the most important predictors of persistent alopecia were V_40Gy_ and V_43Gy_ (Table 7).

All these data taken together indicate that, although low doses (i.e., 16–20 Gy), are critical for acute alopecia (that is likely to recover within some months), higher doses (i.e., 40–43 Gy) are crucial for persistent alopecia.

To our knowledge, the only existing dosimetric study finding a dose-response relationship that described the probability of alopecia after photon-based radiotherapy has been reported in 2004 by Lawenda et al. (25). The authors retrospectively reviewed 26 patients and they concluded that follicle doses of 43 Gy are associated with a 50% risk of permanent alopecia. Their results are notably different from our findings due to two main reasons. First, the authors provided a very rough estimate of the follicle dose, based on the sum of the entrance and exit doses for each contributing radiation field, according to a formula that took into account the absolute dose delivered to the isocenter for the radiation field of interest; by contrast, the present study provided an accurate calculation of the dose to the scalp using a dose-volume histogram analysis calculated by the treatment planning system. Secondly and even more importantly, the patients included in the study from Lawenda et al. were treated with simple conventional photon techniques (typical field arrangements included parallel-opposed fields and right-angle field pairs). On the other hand, all the patients in our series were treated with VMAT-technique. The numerous beam angles and resultant highly conformal dose distributions of intensity-modulated treatments (IMRT and VMAT) make these modalities particularly suited to scalp dose reduction. The use of arcs, typical of the VMAT technique, may further minimize the high doses to the scalp because the surface dose is distributed over the length of the arc (19). The investigation of Penoncello et al. (26) confirmed that VMAT may be superior in minimizing dose to the scalp than static-field IMRT.

The possibility to reduce the dose to the scalp with IMRT techniques has been extensively explored in patients treated WBRT for brain metastases (10–12, 19, 27). These studies differ from the present study for several reasons: firstly, the number of patients included was significantly lower (range 6(12)−17(27) patients) compared to our experience; secondly, the prescription dose for WBRT (EQD2 28–36 Gy) is significantly different than the one used for primary tumors (EQD2 50, 4–60 Gy). Thirdly, most of them did not include clinical data on alopecia: plans of patients who had been previously treated with conventional opposed lateral fields were simply replanned with IMRT to confirm the potential advantage of IMRT techniques in reducing scalp dose (10, 11, 28–30). Lastly, although some series including clinical evaluation of alopecia exist (12, 27, 28), their authors did not generate hypotheses about dose/permanent hair loss relationship and they did not provide clear dose constraints to minimize the risk of chronic alopecia.

Due to the very superficial location of the scalp, the existing uncertainty in the superficial dose calculation deserves some considerations. The accuracy of dose modeling in the build-up region mainly depends on the dose calculation algorithm used in a specific treatment planning system (TPS) (31). MC simulations have been used as a reference tool for superficial dosimetry evaluation of dose calculation algorithms in the commercially available TPS (32, 33) because they were shown to be consistent with measurements obtained by extrapolation chambers (34, 35). To our knowledge, there are no published studies specifically evaluating the accuracy of dose calculation in the build-up region for Monaco TPS. However, since Monaco TPS uses a MonteCarlo algorithm, we can assume that superficial dose is estimated by this TPS with reasonable accuracy.

In this clinical experience, the majority of patients (95%) presented acute alopecia in a wide area of the scalp (by average 30.2%). This phenomenon is due to the fact the highly conformal dose distribution achieved with VMAT comes with the cost of a larger volume of normal tissue receiving low radiation doses that are sufficient to cause an acute injury to the hair bulbs.

On the other hand, VMAT led to satisfying results in terms of hair regrowth (actuarial recovery rate = 98.1% at 18 months after the end of radiotherapy) because of the high conformality and rapid dose fall-off. We believe that the application of our dosimetric findings may further decrease the risk of radiation-induced hair-loss: maintaining V_40Gy_ < 5.4 and V_43Gy_ < 2.2 cc may help in reducing the risk of radiation-induced chronic alopecia. Since these two variables were found to be interdependent, considering the AUC and statistical significance at the ROC analysis, we would suggest to try to meet preferably the specified constraints for V40 in order to minimize the risk of chronic alopecia. Noteworthy, the importance of V40 was confirmed also by the multivariate analysis.

Time to recovery was related to the dose, as shown by Kaplan–Meier analysis and Cox regression analysis that confirmed the significant impact of the dichotomous dosimetric variables on recovery probability during the follow-up. Furthermore, D_0,1cc_ maintained significance also when tested as a continuous variable (*p* = 0,001) at the univariate Cox regression analysis.

Age ≤ 14 was the only clinical factor to be significantly associated with a greater probability of recovery. Younger age was identified as a positive factor also in the series of Rogers et al. (36).

In our experience, chemotherapy was not related to a higher risk of alopecia. Of note, the majority of chemotherapy-treated patients in the present series received temozolomide, whereas, in other experiences where this relationship was found, other drugs with a stronger alopecia-inducing power were used (10, 25). Notably, increased risk due to smoking history was not evidenced in our series.

Another point to mention is that the definition of dosimetric thresholds for chronic alopecia may also help in estimating the risk of this relevant side effect when discussing the toxicity of treatment with our patients. So far, indeed, the scarcity of available data about radiation-induced hair loss has led to great difficulties in providing risk estimates for given doses when radiation treatment is discussed with patients (37).

### Keypoints and Pitfalls of the Study

Strengths of this study are the following: first, given the little literature on possible predictors of radiation induced alopecia in patients treated with photons, this study adds new information, especially considering the fact that it concerns VMAT technique. Secondly, to our knowledge, this is the first existing observational study with detailed measurements of the endpoint on patients treated with photons. On the other hand, our study has several limitations: the lack of a validation cohort to confirm our dosimetric results is probably the most important shortcoming. Secondly, a quality-of-life assessment or a patient-reported outcome data to describe how the patients psychologically experienced the hair loss would have added value to our research. Thirdly, an important drawback of our work is the lack of a more advanced modeling to robustly predict the risk of radiation induced alopecia. In this regard, a very recent study (38) provided normal tissue complication probability (NTCP) model for alopecia in patients treated with scanning beam protontherapy. Although it is necessary to take into account the different dose distribution in the superficial tissues for protons (which makes their results not applicable to photon-based radiotherapy), it is of interest to know that relative scalp surface receiving 21 Gy (S_21Gy_) and age were selected as predictive factors for acute G2 alopecia whereas D_2%_ (near maximum scalp dose) was found to be related to permanent G2 alopecia.

## Conclusions

We recommend contouring the scalp and including it into the organs at risk list.

According to our results, the steep gradient typical of VMAT gives the possibility to limit the volume of the scalp that receives higher doses that are associated with a greater risk of chronic G2-alopecia. At the same time, by using VMAT, a great proportion of the scalp volume will receive low doses that are sufficient to cause acute but transient alopecia in the majority of patients.

Our study provided new constraints for the scalp to use during the inverse planning process that may help in reducing the probability of hair-loss. Once a treatment is planned, these dose thresholds may help also in estimating the risk of alopecia for each single case. Future developments of our research may provide a validation cohort to confirm further improvement in terms of alopecia-free survival.

## Data Availability Statement

The datasets generated for this study are available on request to the corresponding author.

## Ethics Statement

Ethical review and approval was not required for the study on human participants in accordance with the local legislation and institutional requirements. Written informed consent to participate in this study was provided by the participants' legal guardian/next of kin. Written informed consent was obtained from the individual(s) for the publication of any potentially identifiable images or data included in this article.

## Author Contributions

SS and GS conceived the study. SS was in charge of overall direction and planning. MP, FT, MT, GC, ML, EO, CD, RG, and VC performed the contouring of the cases at the treatment planning system and were responsible for the clinical evaluation of the alopecia at the end of the treatment and during the follow-up. CT, LM, and SP were responsible for the plans. SS processed the experimental data. CS performed the statistical analysis. SS wrote the manuscript with inputs from DG, PB, BD, MM, ID, GF, VD, CT, LM, SP, and LL. All authors discussed the results and contributed to final manuscript.

### Conflict of Interest

The authors declare that the research was conducted in the absence of any commercial or financial relationships that could be construed as a potential conflict of interest.

## References

[1] Freites-Martinez A Shapiro J Goldfarb S Nangia J Jimenez JJ Paus R. Hair disorders in patients with cancer. J Am Acad Dermatol. (2019) 80:1179–96. 10.1016/j.jaad.2018.03.055 29660422 PMC6186204

[2] Irvine L Jodrell N. The distress associated with cranial irradiation: a comparison of patient and nurse perceptions. Cancer Nurs. (1999) 22:126–33 10.1097/00002820-199904000-00004 10217028

[3] Steinmann D Paelecke-Habermann Y Geinitz H Aschoff R Bayerl A Bölling T. Prospective evaluation of quality of life effects in patients undergoing palliative radiotherapy for brain metastases. BMC Cancer. (2012) 12:283. 10.1186/1471-2407-12-283 22780988 PMC3434068

[4] Haider M Hamadah I Almutawa A. Radiation- and chemotherapy- induced permanent alopecia: case series. J Cutan Med Surg. (2013) 17:55–61. 10.2310/7750.2012.12033 23364152

[5] Munro AJ Biruls R Griffin AV Thomas H Vallis KA. Distress associated with radiotherapy for malignant disease: a quantitative analysis based on patients perceptions. Br J Cancer. (1989) 60:370–4. 10.1038/bjc.1989.287 2789944 PMC2247172

[6] Lee NY Terezakis SA Intensity-modulated radiation therapy. J Surg Oncol. (2008) 97:691–6. 10.1002/jso.21014 18493919

[7] Gondi V Tomé WA Mehta MP. Why avoid the hippocampus? A comprehensive review. Radiother Oncol. (2010) 97:370–6 10.1016/j.radonc.2010.09.013 20970214 PMC2997490

[8] Beddok A Faivre JC Coutte A Guévelou JL Welmant J Clavier JB. Practical contouring guidelines with an MR-based atlas of brainstem structures involved in radiation-induced nausea and vomiting. Radiother Oncol. (2019) 130:113–20. 10.1016/j.radonc.2018.08.003 30172454

[9] Lee WS Ro BI Hong SP Bak H Sim WY Kim DW. A new classification of pattern hair loss that is universal for men and women: basic and specific (BASP) classification. J Am Acad Dermatol. (2007) 57:37–46. 10.1016/j.jaad.2006.12.029 17467851

[10] Roberge D Parker W Niazi TM Olivares M Treating the contents and not the container: dosimetric study of hair-sparing whole brain intensity modulated radiation therapy. Technol Cancer Res Treat. (2005) 4:567–70. 10.1177/153303460500400510 16173827

[11] Mancini BR Wilkinson JB Kim LH Shaitelman SF Yan D Ionascu D Intensity-modulated radiation therapy or volumetric-modulated arc therapy to reduce alopecia, xerostomia, and otitis after whole brain radiation therapy for brain metastases: a planning analysis. J Radiat Oncol. (2013) 2:177–83. 10.1007/s13566-013-0090-y

[12] Mahadevan A Sampson C LaRosa S Floyd SR Wong ET Uhlmann EJ. Dosimetric analysis of the alopecia preventing effect of hippocampus sparing whole brain radiation therapy. Radiat Oncol. (2015) 10:245 10.1186/s13014-015-0555-9 26611656 PMC4662000

[13] Scoccianti S Detti B Gadda D Greto D Furfaro I Meacci F. Organs at risk in the brain and their dose-constraints in adults and in children: a radiation oncologist's guide for delineation in everyday practice. Radiother Oncol. (2015) 114:230–8. 10.1016/j.radonc.2015.01.016 25701297

[14] Metz CE. Basic principles of ROC analysis. Semin Nucl Med. (1978) 8:283–98. 10.1016/S0001-2998(78)80014-2 112681

[15] Zweig MH Campbell G. Receiver-operating characteristic (ROC) plots: a fundamental evaluation tool in clinical medicine. Clin Chem. (1993) 39:561–77. 10.1093/clinchem/39.4.561 8472349

[16] Pinnix CC Cella L Andraos TY Ayoub Z Milgrom SA Gunther J. Predictors of hypothyroidism in hodgkin lymphoma survivors after intensity modulated versus 3-dimensional radiation therapy. Int J Radiat Oncol Biol Phys. (2018) 101:530–40. 10.1016/j.ijrobp.2018.03.003 29681481 PMC7189963

[17] Cella L Conson M Caterino M De Rosa N Liuzzi R Picardi M. Thyroid V30 predicts radiation-induced hypothyroidism in patients treated with sequential chemo-radiotherapy for hodgkin's lymphoma. Int J Radiat Oncol Biol Phys. (2012) 82:1802–8. 10.1016/j.ijrobp.2010.09.054 21514076

[18] Youden WJ. An index for rating diagnostic tests. Cancer. (1950) 3:32–5. 10.1002/1097-0142 15405679

[19] De Puysseleyr A Van De Velde J Speleers B Vercauteren T Goedgebeur A Van Hoof T. Hair-sparing whole brain radiotherapy with volumetric arc therapy in patients treated for brain metastases: dosimetric and clinical results of a phase II trial. Radiat Oncol. (2014) 9:170. 10.1186/1748-717X-9-170 25074394 PMC4118657

[20] Olsen E Anagen hair-loss: radiation. In: Olsen EA, editor. Disorders of Hair Growth: Diagnosis and Treatment. New York, NY: McGraw Hill; Libraries Australia (1994). p. 225–226

[21] Vaccaro M Guarneri F Brianti P Cannavò SP. Temporary radiation-induced alopecia after embolization of a cerebral arteriovenous malformation. Clin Exp Dermatol. (2015) 40:88–90. 10.1111/ced.12472 25284057

[22] Seol JE Kim DH Park SH Cho GJ Kim H Three cases of radiation-induced temporary alopecia with hair microscopic examination: “coudability hair” might not be specific for alopecia areata. Int J Trichology. (2018) 10:40–3. 10.4103/ijt.ijt_74_17 29440860 PMC5803854

[23] Archambeau JO Pezner R Wasserman T. Pathophysiology of irradiated skin and breast. Int J Radiat Oncol Biol Phys. (1995) 31:1171–85. 10.1016/0360-3016(94)00423-I 7713781

[24] Rosenthal DI Chambers MS Fuller CD Rebueno NC Garcia J Kies MS. Beam path toxicities to non-target structures during intensity-modulated radiation therapy for head and neck cancer. Int J Radiat Oncol Biol Phys. (2008) 72:747–55. 10.1016/j.ijrobp.2008.01.012 18455324 PMC9627578

[25] Lawenda BD Gagne HM Gierga DP Niemierko A Wong WM Tarbell NJ. Permanent alopecia after cranial irradiation: dose-response relationship. Int J Radiat Oncol Biol Phys. (2004) 60:879–87. 10.1016/j.ijrobp.2004.04.031 15465206

[26] Penoncello GP Ding GX. Skin dose differences between intensity-modulated radiation therapy and volumetric-modulated arc therapy and between boost and integrated treatment regimens for treating head and neck and other cancer sites in patients. Med Dos. (2016) 41:80–6. 10.1016/j.meddos.2015.09.001 26764180

[27] Kao J Darakchiev B Conboy L Ogurek S Sharma N Ren X. Tumor directed, scalp sparing intensity modulated whole brain radiotherapy for brain metastases. Technol Cancer Res Treat. (2015) 14:547–55. 10.7785/tcrt.2012.500426 24750002

[28] Ting J Thomas CR McClure JA Scarbrough TJ “Alopecia-less” whole brain radiotherapy (WBRT) via IMRT: preliminary experience and outcomes. Int J Radiat Oncol Biol Phys. (2005) 63(Suppl. 1):S263–4. 10.1016/j.ijrobp.2005.07.451

[29] Witek M Vahknenko Y Siglin J Harrison A Xiao Y Lui H. Dose reduction to the scalp with hippocampal sparing is achievable with intensity modulated radiotherapy. Int J Med Phys Clin Eng Radiat Oncol. (2014) 3:176–82. 10.4236/ijmpcero.2014.33023 29963335 PMC6020831

[30] Pokhrel D Sood S Lominska C Kumar P Badkul R Jiang H. Potential for reduced radiation-induced toxicity using intensity-modulated arc therapy for whole-brain radiotherapy with hippocampal sparing. J Appl Clin Med Phys. (2015) 16:131–41. 10.1120/jacmp.v16i5.5587 26699321 PMC5690185

[31] Panettieri V Barsoum P Westermark M Brualla L Lax I AAA and PBC calculation accuracy in the surface build-up region in tangential beam treatments. Phantombreast case study with the MonteCarlo code PENELOPE. Radiother Oncol. (2009) 93:94–101. 10.1016/j.radonc.2009.05.010 19541380

[32] Cao Y Yang X Yang Z Qiu X Lv Z Lei M Superficial dose evaluation of four dose calculation algorithms. Radiat Phys and Chem. (2017) 137:23–8. 10.1016/j.radphyschem.2016.02.032

[33] Wang L Cmelak AJ Ding GX. A simple technique to improve calculated skin dose accuracy in a commercial treatment planning system. J Appl Clin Med Phys. (2018) 19:191–7. 10.1002/acm2.12275 29411506 PMC5849836

[34] Abdel-Rahman W Seuntjens JP Verhaegen F Deblois F Podgorsak EB. Validation of montecarlo calculated surface doses for megavoltage photon beams. Med Phys. (2005) 32:286–98. 10.1118/1.1829401 15719980

[35] Devic S Seuntjens J Abdel-Rahman W. Accurate skin dose measurements using radiochromic film in clinical applications. Med Phys. (2006) 33:1116–24. 10.1118/1.2179169 16696489

[36] Rogers S Donachie P Sugden E Sharpe G English M Robinson K Comparison of permanent hair-loss in children with standard risk PNETS of the posterior fossa following radiotherapy alone or chemotherapy and radiotherapy after surgical resection. Pediatr Blood Cancer. (2011) 57:1074–6. 10.1002/pbc.22992 21744477

[37] Shakespeare TP Dwyer M Mukherjee R Yeghiaian-Alvandi R Gebski V. Estimating risks of radiotherapy complications as part of informed consent: the high degree of variability between radiation oncologists may be related to experience. Int J Radiat Oncol Biol Phys. (2002) 54:647–53. 10.1016/S0360-3016(02)02996-6 12377314

[38] Palma G Taffelli A Fellin F D'Avino V Scartoni D Tommasino F. Modelling the risk of radiation induced alopecia in brain tumor patients treated with scanned proton beams. Radiother Oncol. (2019) 144:127–34. 10.1016/j.radonc.2019.11.013 31805517

